# Long-Term Melatonin Therapy for Adolescents and Young Adults with Chronic Sleep Onset Insomnia and Late Melatonin Onset: Evaluation of Sleep Quality, Chronotype, and Lifestyle Factors Compared to Age-Related Randomly Selected Population Cohorts

**DOI:** 10.3390/healthcare6010023

**Published:** 2018-03-02

**Authors:** Tom C. Zwart, Marcel G. Smits, Toine C.G. Egberts, Carin M.A. Rademaker, Ingeborg M. van Geijlswijk

**Affiliations:** 1 Faculty of Veterinary Medicine, Pharmacy Department, Utrecht University, Utrecht 3584 CM, The Netherlands; tomczwart@gmail.com; 2 Utrecht Institute for Pharmaceutical Sciences (UIPS), Department of Pharmacoepidemiology and Clinical Pharmacology, Faculty of Science, Utrecht University, Utrecht 3584 CG, The Netherlands; A.C.G.Egberts@uu.nl; 3 Department of Sleep-wake disorders and Chronobiology, Gelderse Vallei Hospital, Ede 6716 RP, The Netherlands; smitsm@zgv.nl; 4 Governor Kremers Centre, Maastricht University, Maastricht 6229 GR, The Netherlands; 5 Department of Clinical Pharmacy, Division of Laboratory and Pharmacy, University Medical Centre Utrecht, Utrecht 3584 CX, The Netherlands; C.Rademaker@umcutrecht.nl

**Keywords:** melatonin, children, CSOI, long-term, efficacy, safety

## Abstract

The extent of continuance of melatonin therapy initiated in pre-pubertal children with chronic sleep onset insomnia (CSOI) was investigated in young adult life. Sleep timing, sleep quality, adverse events, reasons for cessation of therapy, and patient characteristics with regard to therapy regimen, chronotype and lifestyle factors possibly influencing sleeping behavior were assessed. With an online survey using questionnaires (Pittsburgh Sleep Quality Index, Insomnia Severity Index, Morningness-Eveningness Questionnaire, and Munich Chronotype Questionnaire), outcomes were measured and compared with age-related controls. These controls were extracted from published epidemiological research programs applying the same questionnaires. At the moment of the survey, melatonin was still continued by 27.3% of the patients, with a mean treatment duration of 10.8 years. The overall average treatment duration was 7.1 years. Sleep quality of both discontinued and persistent melatonin users did not deviate from controls. Sleep timing and chronotype scores indicated evening type preference in all responders. Adverse events were scarce but the perceived timing of pubertal development suggested a tendency towards delayed puberty in former and current users of melatonin. This study may underestimate the number of children that are able to stop using melatonin due to the response rate (47.8%) and appeal for continuing users. Sleep timing parameters were based on self-reported estimates. Control populations were predominantly students and were of varying nationalities. The statistical power of this study is low due to the limited sample size. Melatonin therapy sustained for 7.1 years does not result in substantial deviations of sleep quality as compared to controls and appears to be safe. The evening type preference suggests a causal relation with CSOI. This study shows that ten years after initiation of treatment with melatonin for CSOI, approximately 75% of the patients will have normal sleep quality without medication.

## 1. Introduction

Since the introduction of melatonin as a treatment option for children with chronic sleep onset insomnia (CSOI), efficacy issues and safety concerns have been debated. Recently, the publication of clinical recommendations [1] and guidelines [2] for the use of melatonin in children renewed this debate on safety [3,4]. Melatonin is widely used as an over-the-counter (OTC) dietary food supplement in Europe and the USA. Many healthcare workers and parents perceive the absence of adverse events reports as confirmation that melatonin is safe. However, others remain skeptical as information on the effectiveness and safety of long-term melatonin therapy is still limited. Although studies in small populations over limited periods of time have shown persistent effectiveness on sleep timing and no alarming adverse effects [5,6,7,8], more data on melatonin therapy over longer periods of time are needed to confirm these findings and to answer the question of what patient characteristics may predict duration of melatonin therapy.

The current study comprises an evaluation of a group of Dutch adolescents and young adults who started melatonin therapy during early childhood for CSOI 9–12 years ago. Primary objectives are (1) to evaluate (dis)continuance of melatonin therapy, actual sleep timing and actual sleep quality on average ten years after treatment initiation, and (2) to investigate the occurrence of adverse events and reasons for melatonin cessation. Secondary objectives are to assess patient characteristics with regard to (1) treatment features and attitudes, (2) chronotype and (3) lifestyle factors that might interfere with endogenous and exogenous melatonin pharmacokinetics, thereby possibly affecting effectiveness of melatonin therapy, long-term treatment outcomes, and the need for continuance of therapy. This study compares former and persistent melatonin users to age-related controls. 

## 2. Materials and Methods

### 2.1. Study Design

All 69 children that finished the melatonin dose finding (Meldos) trial [9], which was conducted between February 2004 and May 2007, were invited to participate in this follow-up study. The study consisted of an electronic, online survey with questions regarding demographics, melatonin therapy features and attitudes, timing of puberty development, lifestyle factors and four validated international questionnaires evaluating sleep timing, sleep quality and chronotype. Last known home addresses of all Meldos participants were provided by Gelderse Vallei Hospital Ede and additional contact information of participants’ parents was available from the Meldos trial and a previous follow-up study [7]. All eligible participants were contacted by telephone, e-mail, postal mail or Facebook. Respondents who agreed to participate in the study were sent an e-mail containing a personal, unique web link providing them access to our online questionnaire. The study protocol was categorized as research not subjected to the Medical Research Involving Human Subjects Act by the Medical Ethics Committee of the University Medical Center Utrecht (UMCU) and approved by the local Research Assessment Committee (BCWO) of the Gelderse Vallei Hospital Ede, both located in The Netherlands. The study was registered in the Netherlands Trial Registry (NTR5930).

### 2.2. Participants

All 69 former participants of the Meldos trial [9] were eligible to participate in this second long-term evaluation. The Meldos trial included children diagnosed with CSOI, aged 6–12, who did not respond to sleep hygiene measures and suffered from sleep onset insomnia for more than four nights per week for more than one year. Sleep onset insomnia was defined as sleep onset later than 20:30 for children aged 6, and for older children 15 min later per year until the age of 12 (22:00) and average sleep onset latency (SOL) exceeding 30 min. Exclusion criteria were CSOI due to psychiatric or pedagogic problems, known intellectual disability, pervasive developmental disorder, chronic pain, known disturbed hepatic or renal function, epilepsy, prior use of melatonin, and use of stimulants, neuroleptics, benzodiazepines, clonidine, antidepressants, hypnotics or beta-blockers within 4 weeks before enrolment.

### 2.3. Control Population

To evaluate the effects of long-term melatonin use in our patient group, and to identify characteristics of patients diagnosed with chronic sleep onset insomnia at a young age, we compared our study group (N = 33) with results found in epidemiological research programs applying the same questionnaires. These populations represent the “normal” population in late 10 s and early 20 s, and vary from N = 154 for the PSQI questionnaire to N = 9500 for smoking habits in the Dutch National Drug Monitoring.

### 2.4. Questionnaire

The questionnaire consisted of eight parts to assess the various aspects regarding patient characteristics, therapy effectiveness, and safety associated with melatonin treatment.

### 2.5. Patient Characteristics

#### 2.5.1. Demographics and Melatonin Use

Gender, age, weight, length, marital status, offspring and highest level of education were registered. Melatonin treatment features and attitudes were evaluated by assessing (dis)continuation of melatonin use, treatment duration, current dose, therapy habits like temporary discontinuation of therapy (drug holidays) and reasons for final cessation of therapy.

#### 2.5.2. Chronotype

Chronotype was assessed with the Kerkhof version of the Morningness-Eveningness Questionnaire (MEQ) and a Dutch version of the Munich Chronotype Questionnaire (MCTQ). The MEQ is a nineteen item, self-administered questionnaire which differentiates between morning- and evening type, first published by Horne & Östberg in 1976 [10,11]. It has been validated in various languages and populations and is widely used for chronotype evaluation [12]. In 1984, a Dutch shortened version of the MEQ was published by Kerkhof [13]. The Kerkhof MEQ consists of seven items, five of which are scored on a 1–4 scale. The remaining questions are scored on a time scale. Total score ranges from 7–31, which is interpreted as follows: ‘7–10’ = definitely evening type, ‘11–14’ = moderate evening type, ’15–21’ = neither type, ’22–25’ = moderate morning type and ’26–31’ = definitely morning type. It was validated in a population consisting of mainly students (N = 275) with an average age of 22.7 years [13]. The Kerkhof MEQ was implemented in our questionnaire since it is simpler than the original MEQ and has shown comparable validity [10,13].

The MCTQ evaluates sleep timing on both working- and non-working days with regard to bedtime, time till lights out, SOL, wake time and time till rise [14]. The MCTQ has been validated with sleep logs, actimetry, Dim Light Melatonin Onset (DLMO) and the MEQ and has shown high correlations [15,16,17]. In this study, a Dutch version of the MCTQ was used which is available on the Ludwig Maximilian University München website [18]. With the results of the MCTQ the midpoint between sleep onset time (SOT) and wake-up time is calculated, which is defined as midsleep on free days (MSF) [19]. The MSF is strongly correlated with DLMO and is an indicator for chronotype [14,20]. Additionally, the MSF was corrected for workday derived sleep debt resulting in the MSFSC, as described by Roenneberg et al. in 2004 [21].

#### 2.5.3. Lifestyle Factors

Smoking habits were evaluated with three items from the smoking section of the Dutch Health Survey (DHS) 2014 [22]. Current smoking status (smoker versus non-smoker), type of tobacco product and smoking frequency were assessed. 

Average daily caffeine consumption was evaluated with three food frequency questions on the average daily consumption of coffee, tea and caffeinated energy drinks over the past twelve months. These food frequency data were multiplied with the caffeine content of a standardized portion of the respective beverage types, as adopted from the Netherlands Nutrition Centre [23].

The use of electronic devices at bedtime was evaluated with three questionnaire items: (1) whether the participant owned a smartphone or tablet, (2) whether the device was brought into the bedroom at bedtime and (3) how often it was used after lights out. Options for the latter question were ‘never’, ‘less than half of the time’, ‘more than half of the time’ or ‘always’.

### 2.6. Therapy Effectiveness

#### 2.6.1. Sleep Quality

Sleep quality was examined with the Pittsburgh Sleep Quality Index (PSQI), adapted for the Dutch population as provided by eProvide^®^. The PSQI is a self-rated nineteen item questionnaire to assess sleep quality and sleep disturbances over the past month [24]. It addresses seven items of sleep: subjective sleep quality, SOL, sleep duration, habitual sleep efficiency, sleep disturbances, use of sleep medication and daytime dysfunction. Each item is weighted on a 0–3 scale. Item scores are summed up to yield the global PSQI score which ranges from 0–21, higher scores indicating worse sleep quality. A cut-off value of greater than five is defined to distinguish between good and poor sleepers.

Insomnia severity was evaluated with the Insomnia Severity Index (ISI), adapted for the Dutch population as provided by eProvide^®^. The ISI encompasses a self-rated, seven item questionnaire to determine the participants’ perception of his or her insomnia severity [25,26]. The ISI evaluates subjective symptoms and consequences of insomnia over the past two weeks in seven items of insomnia: severity of sleep onset and sleep maintenance difficulties, satisfaction with the current sleep pattern, interference with daily functioning, noticeability of impairment attributed to the sleep problem and the degree of distress or concern caused by the sleep problem. Items are scored on a five-point scale from 0 (none) to 4 (very severe), which are summed to a total score of 0–28. Total ISI-score is interpreted as follows: ‘0–7’ = no clinically significant insomnia, ‘8–14’ = subthreshold insomnia, ‘15–21’ = moderate insomnia, ‘22–28’ = severe insomnia.

#### 2.6.2. Sleep Timing

Sleep timing, bedtime, time till lights out, SOL, wake up time and time till rise after wake up were assessed with the MCTQ. With these parameters SOT, rise time (RT) and total sleep time (TST) were calculated.

#### 2.6.3. Safety

Occurrence of therapy associated adverse events like headaches (explicitly solicited) or other adverse events, occurrence of rebound sleep disturbances after (temporary) cessation of therapy, the use of co-medication and perceived timing of pubertal development, were evaluated. Perceived pubertal timing was evaluated with one questionnaire item, by which participants were asked to indicate whether they felt their timing of pubertal development was any earlier or later than most other boys or girls of the same age. Options were ‘much earlier’, ‘somewhat earlier’, ‘about the same’, ‘somewhat later’ or ‘much later’. This questionnaire item was derived from the Puberty Development Scale (PDS) [27].

### 2.7. Statistical Analysis

All data were analyzed in IBM SPSS Statistics 23. Independent sample T-tests were performed in Microsoft Excel 2016. Data on sleep timing, MEQ, PSQI, ISI and lifestyle factors were missing from one participant from the group that had discontinued therapy, this group is referred to as the melatonin treatment discontinuation (STOP) group. Data on occurrence of headache were missing from seven participants from the STOP group. Due to the low number of the persistent users—this group is referred to as the melatonin treatment continuation (CONT) group—statistical analysis was performed for the comparison of young adults using melatonin during their childhood (this study = STOP+CONT) versus the randomly selected age-related population in several cohort studies. Differences within this study between STOP and CONT group are limited to descriptive analysis.

## 3. Results

### 3.1. Patient Characteristics

**Figure 1:**
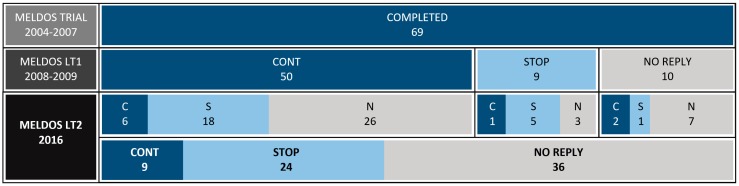
Melatonin continuation, melatonin discontinuation and non-response during the Meldos trial, the Meldos LT1 and the Meldos LT2. CONT/C: continuation; STOP/S: discontinuation; N: no reply.

33 of the 69 former Meldos participants responded (response rate: 47.8%), 32 of which were complete. One responder did not complete the ISI, PSQI, MEQ and MCTQ questionnaires. Nine respondents (27.3%) still used melatonin after an average treatment duration of 10.8 years: CONT group. Twenty-four respondents (72.7%) had discontinued therapy: STOP group. The pattern of melatonin continuation, discontinuation and non-response during the Meldos trial, the first long-term evaluation (Meldos LT1) and the current study (Meldos LT2) are schematically depicted in Figure 1. 

**Figure 2:**
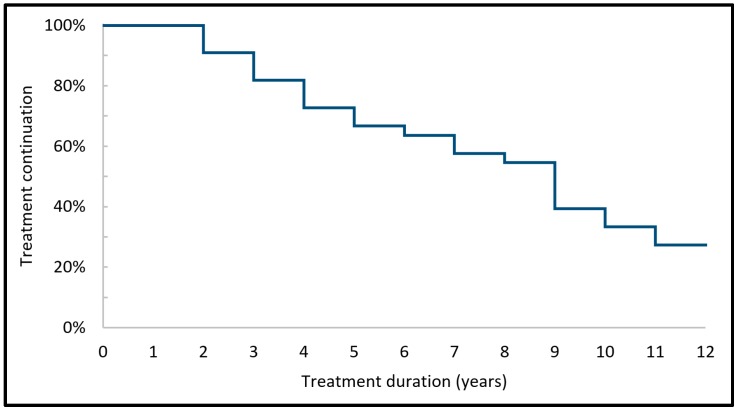
Melatonin treatment continuation of the 33 participants over the years.

The melatonin treatment continuation over the years of the 33 participants of the current study is schematically depicted in Figure 2.

Demographics and treatment features in the total population and specified to the CONT and STOP groups are shown in Table 1. With regard to education, responses indicating higher professional, pre-university and university education were combined and designated as ‘high education level’. Responses indicating secondary and lower vocational education and elementary school as highest achieved education level were combined and designated as ‘low education level’. 

In the CONT group, four participants had temporarily (>6 months) discontinued therapy, but indicated to have restarted since. Five interrupted therapy during holidays and three skipped medication on a weekly basis during weekends. Reasons for interruption were a delayed sleep rhythm during holidays and weekends and checking the need for continuance of therapy. In the STOP group, twenty-one participants indicated to have adopted a delayed sleep rhythm and no longer needed melatonin to fall asleep at the desired (later) bedtime. Two reported unsatisfactory effects on sleep timing as the reason for discontinuation, and one participant did not remember the reason for discontinuation. 

### 3.2. Chronotype

Timing of midsleep on work (MSW) and free (MSF) days of this study were compared to data from Zavada et al., who reported on sleep timing in a population of 1342 Dutch students aged under 25 [16]. To facilitate comparison to the controls, Kerkhof MEQ scores were converted into Horne-Östberg MEQ scores. The Horne-Östberg MEQ score ranges from 16–86, with categories ‘16–30’ = definitely evening type, ‘31–41’ = moderate evening type, ’42–58’ = neither type, ’59–69’ = moderate morning type and ’70–86’ = definitely morning type [10]. Mean Horne-Östberg MEQ score of our total population was compared to data from Zavada et al. [16]. 

**Figure 3:**
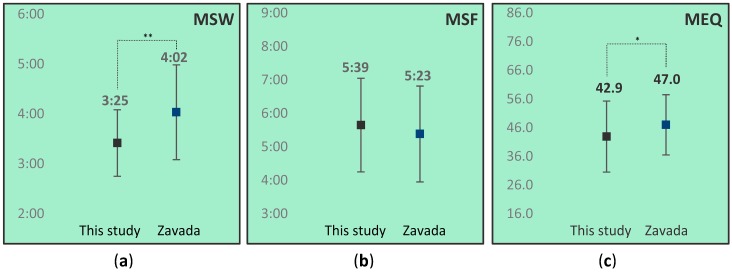
Comparison of mean midsleep on work days (MSW) (**a**), midsleep free days (MSF) (**b**) and Morningness-Eveningness Questionnaire MEQ (**c**) score between this study (N = 32) and data from Zavada et al. (N = 1342) [16]. MSW and MSF in hh:mm. Whiskers represent standard deviations. * *p* < 0.05; ** *p* < 0.01.

Results for MSW, MSF and MEQ score comparisons are depicted in Figure 3.

As is depicted in Figure 3, MSW score, and mean MEQ score of the total population in this study were statistically significant lower than that of controls in the Zavada study, indicating a preference towards eveningness in our population as compared to controls. MSF scores did not differ from the population of 1342 Dutch students. 

**Figure 4:**
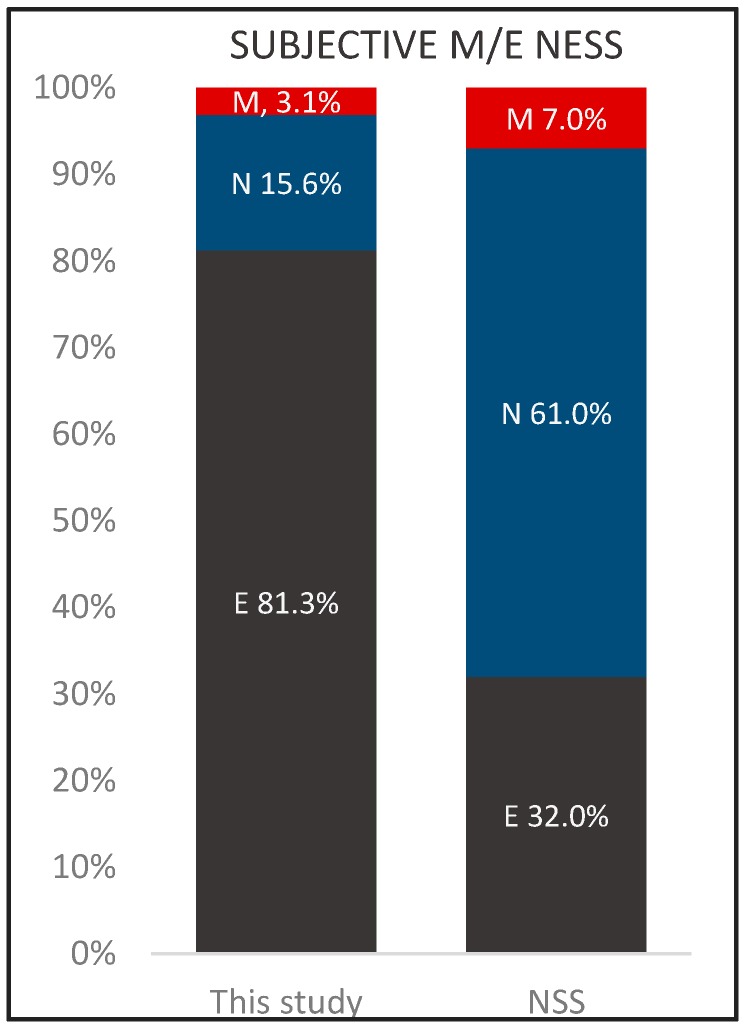
Self-rated morningness-eveningness (M/E-ness) from this study (N = 32) and data from the NSS 2016 (N = 1372) [28]. M: morning type; N: neither type; E: evening type.

Results for this study group with regard to self-rated morningness-eveningness (M/E-ness), as derived from Kerkhof MEQ question item 7, were compared to data from the National Sleep Survey (NSS) 2016. The NSS 2016 reported on the sleep timing of a population of 1372 Dutch students aged 21.7 [28]. Results are depicted in Figure 4. In the control population 61% rated their selves as neither morning nor evening type, as compared to this study population only 15.6%. In this study 81.3% could be categorized as eveningness type, in the control population this was 32%.

### 3.3. Lifestyle Factors

Cigarette smoking prevalence in this study was compared to data from the Netherlands National Drug Monitor (NDM) 2015 report, which reported on smoking prevalence of Dutch 16–20 old’s. 23% of this age group reported to smoke now and again (N = 9500), compared to this study 6 persons, 18.2% [29].

Estimated mean daily caffeine consumption over the past twelve months of this study group was compared to data from the European Food Safety Authority (EFSA) 2015 report on the safety of caffeine, which reported on mean daily caffeine intake in a population of 1142 Dutch adolescents aged 10–17, being 69.5 mg/day [30]. Mean caffeine consumption, was 78.3 mg/day for this study group. 

All respondents possessed a smartphone or tablet and all but one (96.9%), brought the device into the bedroom at bedtime. Results on the use of electronic devices were compared to data from Fossum et al., who reported the use of electronic devices at bedtime at least once a week in a population of 532 Norwegian students aged 22.9 on average was 94.7% [31]. The results of that study refer to the use of any type of electronic device at bedtime (television, computer, gaming console, tablet, mobile phone or audio player), while results from this study refer specifically to the use of a smartphone or tablet at bedtime.

### 3.4. Therapy Effectiveness

#### 3.4.1. Sleep Quality

**Figure 5:**
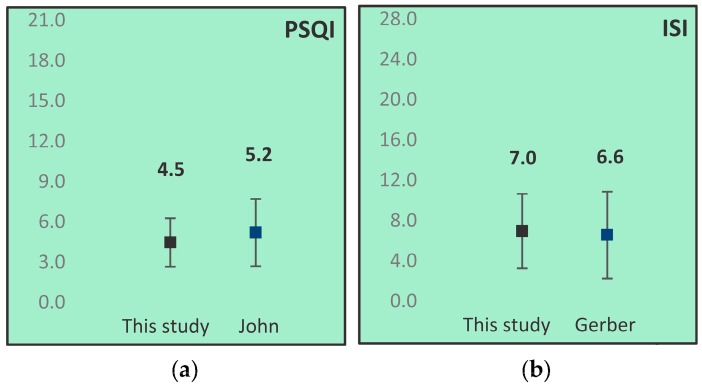
Comparison of mean Pittsburgh Sleep Quality Index (PSQI) (**a**) and Insomnia Severity Index (ISI) (**b**) scores of this study (N = 32) and data from control populations (PSQI: N = 154 [32], ISI: N = 862 [33]). Whiskers represent standard deviations.

PSQI and ISI scores from the CONT and STOP group combined were compared to data from control populations. PSQI scores were compared to data from John et al., who reported on the PSQI scores of a population of 154 Dutch students aged 20.6 on average [32]. ISI scores were compared to data from Gerber et al. [33], who reported on the ISI scores of a population of 862 Swiss students with a mean age of 24.7 years. Results from these comparisons are depicted in Figure 5.

Overall, no differences between the population in this study and age related controls were found for PSQI or ISI scores. The CONT group showed a tendency towards higher mean PSQI and ISI scores than the STOP group (data not shown). Participants with PSQI scores greater than 5 were designated as ‘poor sleepers’ PSQI scores greater than 7 were considered ‘possible insomniacs’. This study group included poor sleepers and no possible insomniacs. Participants with ISI scores 8–14 are considered ‘subthreshold insomnia’. This study group included nine (28.1%) subthreshold insomniacs and no moderate insomniacs, the pathologic PSQI and ISI scores coincide for seven participants, two participants have only one pathologic score. 

#### 3.4.2. Sleep Timing

**Figure 6:**
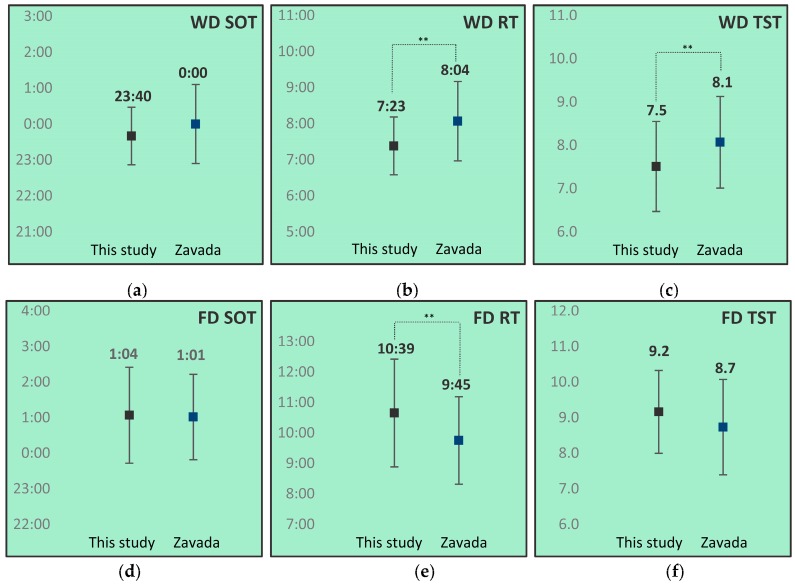
Comparison of work- (**a**–**c**) and free (**d**–**f**) day sleep onset time (SOT) (**a**) and (**d**), rise time (RT) (**b**) and (**e**) and total sleep time (TST) (**c**) and (**f**) between this study group (N = 32) and data from Zavada et al. (N = 1342) [16]. WD: work day; FD: free day. SOT and RT in hh:mm. TST in hours. Whiskers represent standard deviations. * *p* < 0.05; ** *p* < 0.01.

The SOTs, RTs and TSTs for both work- and free days of this study were compared to data from Zavada et al. [16]. Results of these comparisons are depicted in Figure 6.

This study group showed an earlier mean workday RT, shorter workday TST, and later free day RT than controls. 

SOL was also determined in this study group, and compared to data from the NSS 2016 [28]. In the current study, 14 (43.7%) indicated to need over 20 min to fall asleep, mean sleep onset latency (SOL) was 34.7 min. During the previous study 16 (27.1%) reported SOLs of over 20 min, with a mean SOL of 35.1 min [7]. The mean SOL from the NSS 2016 (N = 1372) was 26.0 min [28].

### 3.5. Safety

#### 3.5.1. Adverse Effects

The questionnaire on occurrence of adverse events in was answered by 26 participants in this study. Outcomes on headache and nausea at start were comparable to the previous long-term study, with one third reporting a headache once a month or more, two third seldom or never [7]. One participant reported restless legs and drowsiness.

Eleven participants (39.3%) experienced sleeping difficulties following (temporary) discontinuation. Thirteen participants (39.4%) used co-medication including budesonide, desloratadine, fluticasone, levocetirizine, mebeverine, methylphenidate, miconazole, oral contraceptives, risperidone, ropinirole and salbutamol. 

#### 3.5.2. Pubertal Timing

**Figure 7:**
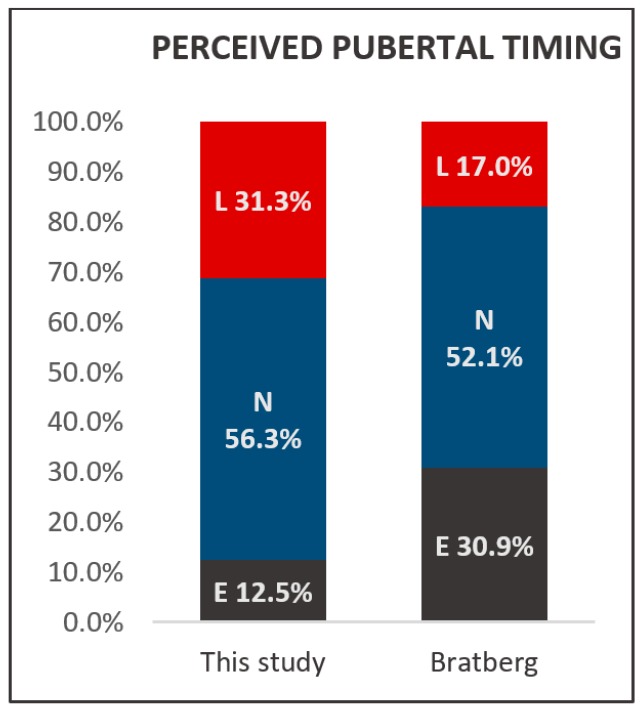
Perceived pubertal timing in this study and from Bratberg et al. (N = 4058) [34]. L: later; N: normal; E; earlier.

With regard to perceived pubertal timing, two participants filled in the option ‘much earlier’. For statistical reasons, the answers ‘much earlier’ and ‘somewhat earlier’ were combined and designated as ‘earlier’. This is also applicable for the option ‘much later’ (N = 1), this answer was combined with the option ‘somewhat later’ and the combination designated as ‘later’. Results were compared with data from Bratberg et al., who reported on perceived pubertal timing in a population of 8951 Norwegian adolescents and young adults aged 13–19 [34]. In the supplementary material, Bratberg et al. stratified pubertal timing results into two age groups: 13–15 and 16–19 year olds. The pubertal timing results of the group of 16–19 year olds (N = 4058) was compared to the Meldos participants. 31.3% of the participants experienced their pubertal timing as late while controls in Bratberg’s study 17.0% denounced their pubertal timing late. Results are depicted in Figure 7.

## 4. Discussion

Of 33 responders, 27.3% (N = 9) still used melatonin after an average treatment duration of 10.8 years. Participants show sleep quality which does not deviate from controls aged 20.6 [32], and comparable insomnia severity as compared to controls. These findings suggest sleep quality and insomnia severity of these former problem sleepers have generally normalized over time possibly facilitated by adopting a postponed sleeping rhythm, whereupon the misalignment of biological and social timing during (pre) puberty is resolved [35]. The problem sleepers in the continuation group use melatonin at higher doses than the other five participants in this group, 3, 4 and twice 5 mg, versus 0.5, 1, 2 and twice 3 mg, without deviations with respect to timing of administration, co-medication or lifestyle factors compared to the other participants from the CONT group. As high melatonin doses may result in spill-over effects [36], the applied melatonin doses might have been too high in these participants, resulting in poor sleep quality and insomnia. SOL from both groups did not deviate from Dutch controls aged 21.7 which suggests the SOL of our population generally normalized since early childhood. 31.3% of the participants experienced their pubertal timing as late which is a larger proportion than the 17.0% of controls in Bratberg’s study that denounced their pubertal timing as late.

Chronotype MEQ score of this study population was significantly lower than seen in controls aged <25. Assessment of subjective M/E-ness showed a distinctively large presence of self-reported evening types in our population as compared to Dutch students aged 21.7 [16]. These findings suggest an inclination to the evening type in our population, suggestive for a causal relationship with CSOI. 

Smoking has been associated with sleep disturbance, resulting in shorter TSTs, longer SOLs and worse sleep quality (higher PSQI scores) for current smokers than former and never smokers [37,38]. On the other hand, fatigue resulting from sleep difficulties and poor sleep quality might stimulate smoking [39]. Additionally, late chronotypes are more often habitual smokers than normal or early chronotypes [40]. Interestingly, smoking prevalence in this study group, which showed an inclination to the late chronotype, was comparable to controls aged 16–20. Caffeine consumption in this study group was comparable to the consumption in a cohort study in Dutch adolescents aged 10–18. Problem sleepers could be prone to consume more caffeine in an attempt to compensate for their daytime sleepiness. Caffeine consumption is associated with sleeping difficulties in adolescents and young adults (shorter sleep duration and increased SOL and wake time after sleep onset), this could add to their sleeping difficulties [41]. Late chronotype is associated with a higher caffeine consumption [40,42] but not in this study group. Combined with the smoking habits observation we speculate that this study group, despite the eveningness preferences, might be more aware of favorable attitudes with regard to healthy sleep conduct. In respect of the use of electronic devices at bedtime, this study group did not seem to deviate from controls aged 22.8 [31]. 

Tobacco smoke [43,44,45] and caffeine [46,47] interfere with CYP1A2 metabolism thus effecting endogenous and exogenous melatonin levels and light emitted from electronic devices suppresses endogenous melatonin levels [48,49,50]. Smoking and higher consumption of caffeine might enhance the need for continuance of therapy, or at least do not support ending therapy. 

Perceived pubertal timing was at about the same time as that of their peers for 50% of our population. These results are consistent with data from controls aged 16–19. The percentage of our population that indicated their timing of pubertal onset occurred later than that of their peers suggested an inclination towards delayed timing of pubertal development. However, one should be careful when interpreting these results, as perceived pubertal timing is only indicative of and not directly related to the actual timing of pubertal development [51]. Also, perceptions about pubertal timing results are known to be dependent on the age of assessment and to vary during maturation [51,52].

Sleep timing parameters of this study group showed earlier RTs and shorter TSTs on work days, and later RTs and longer TSTs on free days than controls aged <25. The combination of short workday TSTs, long free day TSTs and late free day RTs might indicate compensation of workday derived sleep debt, a phenomenon typical for the late chronotype [14,53]. As our population indeed showed an inclination to the evening chronotype, these results were consistent with this. 

During the previous long-term evaluation, Van Geijlswijk et al. reported a mean bedtime of 20:54 (SD 0:44) and mean TST of 9.6 h (SD 0.85) [7]. In the current study, mean bedtime and TST were 23:40 (SD 1:08) and 8.3 h (SD 0.85) respectively. The decline seen in TST with respect to the previous long-term evaluation was consistent with our expectations, as sleep duration gradually decreases in the transition from childhood to adolescence and into early adulthood [54,55]. This decrease in TST during maturation is mainly assigned to later bedtimes and not earlier RTs, which corresponds with our findings. SOL from this study groups did not deviate from Dutch controls aged 21.7 which suggests the SOL of our population had generally normalized (lowered) since early childhood. This finding is in contrast to normative age-related SOL transitions, as SOL generally remains stable during early maturation and (moderately) increases with age after reaching full adulthood [56]. However, these normative age-related SOL transition might not directly apply to our population because of their history of CSOI. During the previous study, SOL was registered as “20 min or shorter” (SOL unspecified) or “longer than 20 min” (SOL specified) [7]. 43 (72.9%) indicated to sleep within 20 min or less, whereas 16 (27.1%) reported SOLs of over 20 min. Mean SOL in the latter group was 35.1 min. In the current study, 14 (43.7%) indicated to need over 20 min to fall asleep, mean SOL was 34.7 min. These results suggest SOL to have generally remained stable since the previous follow-up study.

Consistent with results from the previous long-term evaluation, adverse events were scarce and seemed to be of acceptable nature. Occurrence of headache was comparable between both study groups and not higher than the prevalence of headache amongst various European countries (50%), as reported by Stovner et al. [57]. Reported co-medication included ropinirole, which is authorized for the treatment of restless legs syndrome. As restless legs syndrome has been reported as an adverse effect of melatonin [58], the use of ropinirole might indicate the occurrence of an unreported adverse event here, which however could not be substantiated. 

### Strenghts and Limitations

This study is, to our knowledge, the first to evaluate the effectiveness and safety of pediatric melatonin therapy over more than a decade. Combining information from treatment initiation and outcomes during early childhood and continuance through adolescence and into early adulthood is unique and renders this study very valuable. However, several methodological limitations of the study must be addressed. First, an overestimation of the percentage of melatonin continuation might have occurred, as former participants who still use melatonin could have been more willing to participate in this study than those who discontinued melatonin. This might have resulted in a higher response rate amongst melatonin users as compared to non-users, and, therefore, result in an underestimation of children that are able to stop using melatonin. Secondly, sleep timing parameters were based on self-reported estimates instead of objectively measured with for instance polysomnography or actimetry. Another aspect to be taken into account is that the implemented control populations consisted mainly of students. Student populations deviate from the general population with respect to education level and social obligations and might therefore not be ideal to function as control populations in general. However, as 63.6% of our population was enrolled in or had completed higher education, our population was actually quite closely related to student populations with respect to education level and social obligations. Furthermore, some student populations used for comparison originated from Norway and Switzerland. These populations may deviate from their age-related Dutch peers with respect to sleep habits, which could have influenced some of our findings. And lastly, statistical power of the study was low due to the limited sample size and occurrence of recall bias as a result of the implementation of various retrospective question items could not be ruled out.

## 5. Conclusions

We found that of the children with sleep onset problems related to delayed endogenous melatonin onset treated with exogenous melatonin initiated before the age of twelve, 27% continue melatonin therapy into adulthood. Participants did not show deviations from age-related controls with regard to sleep quality, indicating sleep quality to have normalized over time. In our population the number of the evening chronotype as compared to controls of the same age is significantly higher, suggesting a causal relation with CSOI. More participants perceived pubertal timing as late as compared to controls of the same age. To which extent these subjective results indicate an actual delay in onset of puberty as a result of melatonin treatment in early childhood is inconclusive. Participants in this study showed shorter total sleep time during workdays and more compensation for workday-derived sleep debt during weekends, which is attributable to the prevailing eveningness preference. 

A total 36 of the 69 children prescribed melatonin did not respond to our call. The distribution of STOP and CONT could be different in the non-responders. Our study may underestimate the number of children that are able to stop using melatonin. Sleep timing parameters were based on self-reported estimates. Control populations were predominantly students with varying nationalities. These populations may deviate from our age-related study population. The statistical power of the study was low due to the limited sample size and occurrence of recall bias as a result of the implementation of various retrospective question items cannot be ruled out.

In conclusion, long-term melatonin therapy appeared to be safe after an average of 7.1 years treatment based on data from this limited population. The results of this study indicate that approximately 75% of the children with CSOI who are treated with melatonin will have normal sleep quality without medication ten years later. 

## Figures and Tables

**Table 1:** Demographics and treatment features of the total population (this study) and the CONT and STOP groups.

		This Study	CONT	STOP
N		33	9	24
Males		14 (42.4%)	2 (22.2%)	12 (50.0%)
**Age (years)**	Mean	19.6	20.3	19.4
	Range	16.7 to 23.2	17.6 to 21.9	16.7 to 23.2
	SD	1.9	1.5	2.0
**TD (years)**	Mean	7.1	10.8	5.7
	Range	1.0 to 11.9	9.6 to 11.9	1.0 to 10.9
	SD	3.5	0.8	3.2
**Dose (mg)**	Mean	n/a	2.9	n/a
	Range	n/a	0.5 to 5.0	n/a
	SD	n/a	1.6	n/a
**TOA (hh:mm)**	Mean	n/a	21:46	n/a
	Range	n/a	19:00 to 23:00	n/a
	SD	n/a	1:06	n/a
**BMI (kg/m^2^)**	Mean	21.2	23.1	20.5
	Range	17.0 to 29.8	19.0 to 29.8	17.0 to 26.6
	SD	2.7	3.3	2.1
**Education level**	High	21 (63.6%)	7 (77.8%)	14 (58.3%)
	Low	12 (36.4%)	2 (22.2%)	10 (41.7%)
**Relationship**	Yes	11 (33.3%)	1 (11.1%)	10 (41.2%)
	No	22 (66.7%)	8 (88.9%)	14 (58.8%)
**Offspring**	Yes	1 (3.0%)	0 (0.0%)	1 (4.2%)
	No	32 (97.0%)	9 (100.0%)	23 (95.8%)

TD: treatment duration, TOA: time of administration, BMI: body mass index.
